# Advances in the Study of the Transcriptional Regulation Mechanism of Plant miRNAs

**DOI:** 10.3390/life13091917

**Published:** 2023-09-15

**Authors:** Caixia Teng, Chunting Zhang, Fei Guo, Linhong Song, Yanni Fang

**Affiliations:** College of Horticulture and Forestry Science, Huazhong Agricultural University, Wuhan 430070, China; caixiateng2000@163.com (C.T.); 17684570598@163.com (C.Z.); guofei@mail.hzau.edu.cn (F.G.);

**Keywords:** plant, miRNAs, promoter, transcription regulation, molecular mechanism

## Abstract

MicroRNAs (miRNA) are a class of endogenous, non-coding, small RNAs with about 22 nucleotides (nt), that are widespread in plants and are involved in various biological processes, such as development, flowering phase transition, hormone signal transduction, and stress response. The transcriptional regulation of miRNAs is an important process of miRNA gene regulation, and it is essential for miRNA biosynthesis and function. Like mRNAs, miRNAs are transcribed by RNA polymerase II, and these transcription processes are regulated by various transcription factors and other proteins. Consequently, the upstream genes regulating miRNA transcription, their specific expression, and the regulating mechanism were reviewed to provide more information for further research on the miRNA regulatory mechanism and help to further understand the regulatory networks of plant miRNAs.

## 1. Introduction

Small RNAs, which range in size from 20 to 24 nucleotides, are derived from dsRNAs through processing mediated by the RNase III enzyme [1]. They can be categorized into several groups based on differences in their biogenesis and function [1]. miRNAs and small interfering RNAs (siRNAs) are two major classes of endogenous small RNAs in plants. siRNAs can be subdivided into *trans*-acting siRNAs, repeat-associated siRNAs (rasiRNAs), heterochromatic siRNAs (hc-siRNAs), and nat-siRNAs in plants (nat-siRNAs) [1,2].

**Figure 1:**
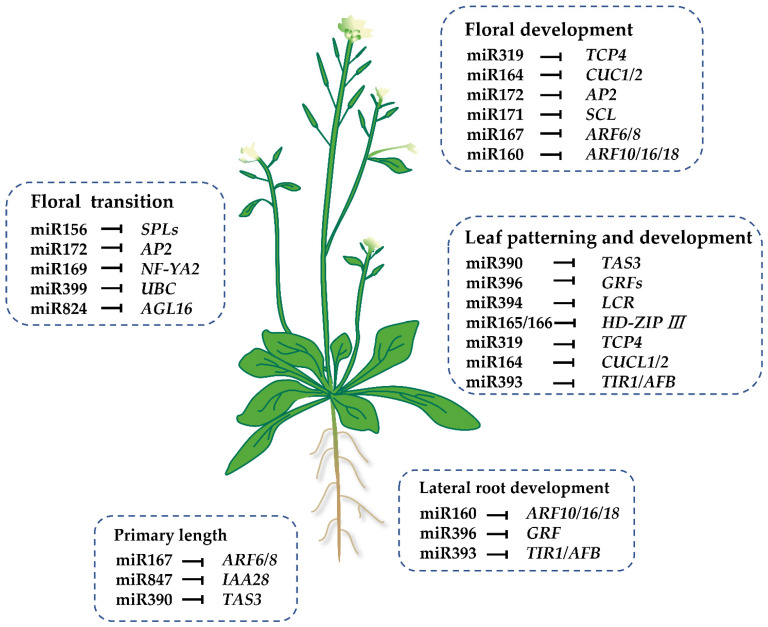
Regulatory functions of miRNAs on plant growth and development. *MYB* (*myeloblastosis oncogene*), *SPL* (*AQUAMOSA promoter-binding protein-like*), *NAC* (*NAM*, *ATAF1/2*, *CUC1/2*), *AP2* (*APETALA2*), *GRF* (*growth-regulating factor*), *ARF* (*auxin-responsive factor*), *CUC* (*cup-shaped cotyledon*), *AGL* (*AGAMOUS-Like*), *HD-ZIP III* (*class III homeodomain leucine zipper*), *UBC* (*ubiquitin-conjugating enzyme*), *TAS* (*trans-acting short-interfering RNA*), *LCR* (*leaf curling responsiveness*), *TCP* (*teosinte branched*), *NF-YA2* (*nuclear transcription factor Y subunit alpha*), *SCL* (*SCARECROW-Like*), *and AFB* (*auxin receptor F box protein*).

Most plant miRNAs originate from intergenic regions and are transcribed from their own promoters [3]. Some other miRNAs originate from non-coding RNAs or the introns of coding genes [3]. Under the action of RNA polymerase II (Pol II), miRNA genes are transcribed into precursor transcript pri-miRNAs in the nucleus. With the help of Dicer enzymes DCL1, pre-miRNAs are cleaved from pri-miRNAs, form into stem–loop structures, and are subsequently cleaved into double-stranded miRNA/miRNA* complexes [3,4]. After the miRNA* is degraded, the mature miRNA binds to the Argonaute1 (AGO1) protein, which forms the RNA-induced silencing complex (RISC) to regulate the expressions of target genes post-transcriptionally through direct transcript cleavage or translation repression [5,6]. To analyze the biological functions and regulatory mechanism mediated by miRNAs, many target genes have been identified using degradome sequencing, and some of them have been validated in vitro or in vivo in recent years. Most target genes of conserved miRNA are transcription factors, such as MYB (myeloblastosis oncogene), SPL (AQUAMOSA promoter-binding protein-like), NAC (NAM, ATAF1/2, CUC1/2), AP2 (APETALA2), the Zinc finger protein HD-ZIP (homeodomain leucine zipper) family, GRF (growth-regulating factor), and ARF (auxin-responsive factor), etc. (Figure 1) [6,7,8,9,10], which make miRNAs key players in the plant regulatory network [11,12]. In addition to transcription factors, some miRNAs target resistance genes [13], ubiquitin-conjugating enzymes, and other genes [14]. Numerous studies have shown that miRNAs play wide and important roles in plant growth and development (Figure 1), the flowering cycle, hormone signal transduction, the stress response, and so on [8,15,16,17,18,19,20,21,22,23].

Transcriptional regulation is important for miRNA expression. Like protein-coding genes, the transcription of miRNA genes is regulated by various transcription factors and other proteins. In general, transcription factors regulate the specific expressions of miRNA genes through binding to the DNA-binding domains and *cis*-acting elements on the miRNA promoters. In addition to transcription factors, some proteins can regulate miRNA transcription by binding to RNA polymerase II to affect the accumulation of polymerase in the miRNA promoter region. It is widely known that miRNAs play significant roles in various processes of plant development by targeting and regulating many genes post-transcriptionally. But how are these miRNAs transcribed from miRNA genes? What studies have been conducted in plants on the upstream regulators of miRNAs? What proteins or transcription factors regulate the transcription of miRNAs to obtain spatiotemporal-specific expression, and how do miRNAs obtain transcription and expression in response to external signals to adapt to environmental changes? To answer these questions, studies on the upstream regulatory factors of miRNAs have been carried out in several plants to analyze the spatiotemporal-specific expressions and transcriptional regulatory mechanisms of miRNAs. Therefore, this review summarizes the progress of the research on the transcriptional regulatory factors of miRNA genes, which will help to deepen our understanding of the regulatory network and molecular mechanisms of miRNAs in the plant lifecycle.

## 2. Identification of Promoters and *cis*-Acting Elements of miRNAs

Many plant miRNA genes are located in intergenic regions as independent transcriptional units. A few miRNAs are located in the intron region of protein-coding genes, which is co-transcribed with the host genes [3,24]. Like coding genes, eukaryotic miRNA genes are transcribed by Pol II under the regulation of general and specific transcription factors [25,26]. The miRNA promoter consists of the core promoter region and distal upstream region. The core promoter region contains elements such as an initiator, TATA box motifs, CAAT box motifs, and *cis*-acting elements. The initiator is a conserved sequence located near the Transcription Start Site (TSS), while the TATA box is a conserved AT-rich sequence about 30 bases upstream of the TSS, which regulates transcription initiation together with the initiator. The CAAT box is located about 85 bases upstream of the TSS and controls the frequency of transcription initiation. The upstream distal region comprises multiple *cis*-acting elements that specifically bind to *trans*-acting factors to synergistically regulate the transcription of miRNAs and the spatiotemporal-specific expressions of miRNAs. *Trans*-acting siRNAs (ta-siRNA) are a class of endogenous small RNAs that are produced from non-coding *TAS* genes. nat-siRNAs are derived from the overlapping transcript of two adjacent genes located on opposite strands [27]. *TAS* genes are transcribed from their own promoter by the RNA polymerase II as long primary RNAs [28]. nat-siRNA biogenesis also relies on the transcription of a pair of antisense genes produced by RNA polymerase II. Therefore, the precursors of ta-siRNAs and nat-siRNAs are transcribed by the Pol II promoter, which is similar to miRNA. Different from the other classes of small RNAs, the precursors of hc-siRNAs are generated by RNA polymerase IV on repetitive regions and transposable elements [29].

miRNA promoter and *cis*-acting element identification is important for transcriptional regulation analyses of miRNAs. Previously, researchers identified 63 miRNA transcriptional start sites in *Arabidopsis* using 5’RACE technology and found that the majority of miRNA promoters contained TATA-boxes [30]. In recent years, the promoters of many miRNA genes have been identified in a variety of plants using multiple bioinformatic prediction methods (Table 1). By applying their self-developed computational sequence-centric method, common query voting (CoVote), Zhou et al. [26] predicted the putative core promoters for the most known intergenic miRNA genes of *Arabidopsis* and rice. TSSP (http://linux1.softberry.com, accessed on 12 September 2023) is a tool that predicts the TSS, combing characteristics describing the functional motifs of common core promoters and the composition of these sites. The promoters of miRNAs can be obtained after TSSs are predicted based on the general rule that the promoter region of each gene is located 1500 bp upstream of the TSS [31]. Using the promoter prediction method developed by Zhou et al. [26], the sequences for the TSS were predicted through the TSSP database after searching the 2000 bp upstream of the 5′ end of the pre-miRNA or the sequences between 400 bp downstream of the neighboring protein-coding gene and the pre-miRNA. By these means, a total of 249 promoter sequences for 158 miRNAs precursors in rice [32], 229 promoters for 139 miRNA precursors in poplar [33], 191 promoters for 122 miRNA loci downloaded in the miRBase, and 64 TSSs for 22 phosphorus-deficient responsive miRNAs in soybean [34,35] have been successfully identified. Additionally, 132 TSSs of 42 miRNA were discovered in *Arabidopsis* using a computational method developed from the genome-wide profiles of nine histone markers, including H3K4me2, H3K4me3, H3K9Ac, H3K9me2, H3K18Ac, H3K27me1, H3K27me3, H3K36me2, and H3K36me3 [36]. A total of 699 promoters and 440 miRNA TSSs have been predicted in soybean using degradome libraries and the TSSP software [37]. In total, 21 high-quality promoters of 23 intergenic miRNAs in cassava were predicted via a hybrid computational method combining PromPredict and the TSSP software based on the DNA free energy change and a common core regulatory element analysis [38]. Another computational sequence-centric method, named the Query-Ranked Frequent Rule (QRFR), was developed by Zhou et al. for identifying the core promoter regions of miRNA genes [39]. In total, 47 core promoters of 40 miRNA genes in *Arabidopsis* studied in [30] were tested using the QRFR, and 34 were correctly confirmed [30,39].

Today, small RNA sequencing and computational prediction technology have been rapidly developed to help with the genome-wide identification of miRNAs and their precursors. Unlike for protein-coding genes, the distance from TSSs to pre-miRNAs is longer and more irregular. Presently, except for model plants such as *Arabidopsis*, the TSSs of the miRNAs in other higher plants are mainly obtained through biotech software prediction due to the high cost of experimental methods. Consequently, the promoter identification of miRNAs in previous publications has usually been limited to beginning upstream from mature or pre-miRNAs due to the lack of the exact TSS information of the miRNAs. This may cause false positives for miRNA promoters. Therefore, more experimental validation is needed to determine the location of the TSSs and promoters of miRNAs in future studies.

In addition to miRNA promoters, numerous *cis*-acting elements of these miRNA promoters have been identified in several species, such as elements regulating plant growth development, hormone response elements, and stress response elements. In the previous study, AtMYC2, ARF, SORLREP3, and LFY transcription-factor-binding site motifs were discovered in *Arabidopsis* to be enriched in miRNA promoters by comparing the promoter elements of 63 miRNA genes and coding genes, as well as randomly selected genomic sequences, using a PWM (Position Weight Matrix) analysis, showing that these transcription factors may be involved in the transcription of *Arabidopsis* miRNAs [40].

PLACE (http://www.dna.affrc.go.jp/htdocs/PLACE, accessed on 12 September 2023) [41] and the PlantCare database (http://bioinformatics.psb.ugent.be/webtools/plantcare/html, accessed on 12 September 2023) [42] are widely used for miRNA promoter *cis*-acting element analyses. In soybean, numerous P-responsive *cis*-elements from the promoters of miRNAs in response to P deficiency were predicted using the PlantCare database [35]. It was found that the frequency of occurrence of the PHR1-binding element, PHO-like-binding element, W-box, and TC element in the promoters of miRNA genes in response to P deficiency (miR156, miR159, miR166, miR167, and miR168, etc.) was higher than that of miRNA genes not responding to P deficiency [35]. In cassava, *cis*-regulatory elements relevant to defense and stress responsiveness, fungal elicitor responsiveness, and hormonal responses were discovered to be abundant in the promoter region of miR160 and miR393 that responds to anthracnose disease, including anaerobic inducible elements (AREs), heat stress response elements (HSE), salicylic acid response elements (TCA-element), TC-rich repeats, fungal inducible response elements (Box-W1), drought-inducible response elements (MBS), and methyl jasmonate response elements (TGACG-motif) [43]. Multiple TC-rich repeats and TCA-elements were also discovered on the promoters of 15 *Arabidopsis* miRNAs responding to *Bacillus velezensis* FZB42 [44].

An analysis of the miRNAs involved in plant salt stress (miR169, miR319, miR393, miR396, and miR398, etc.) in rice revealed that they contained common regulatory elements on their promoters, including GC-boxes, GATA-boxes, MYB response elements, MYC response elements, ABA response elements (ABRE), W-boxes, and zinc finger protein DNA-binding elements (DOF) [45]. Moreover, *cis*-elements for the miRNA genes involved in environmental changes have also been discovered in plants. Environmental SO_2_ is a major air pollutant that has a severe impact on plant growth and development. It was found that the regulatory mechanisms of plant miRNAs in response to SO_2_ stress have similarities with pathogen-mediated stress responses [46]. An analysis of the promoters of 32 differentially expressed miRNAs in response to SO_2_ stress revealed that the fungal-inducer response element Box-W1 and hypoxia response elements (HREs) were more frequently present in the promoters of the SO_2_-stress-responsive miRNAs than in the promoters of other miRNAs [46]. miR397, miR398, and miR408 are copper-deficient responsive miRNAs. To investigate the effect of copper concentration on the expression of miRNAs, in vitro cultured grape seedlings were treated with different copper concentrations for 30 days [47]. The miR397a, miR398a, miR398b/c, and miR408 expressions in the grape leaves and roots decreased with an increasing copper concentration [47]. Subsequently, abundant (6–9) CuRE (GTAC core motif) elements were identified on the promoters of four miRNAs, revealing the molecular mechanism of CuRE elements in the plant response to copper deficiency [47].

In addition, *cis*-elements for miRNA genes have been identified in woody plants as well. Multiple hormone response-related elements were identified in the promoters of 13 miRNAs in rubber tree and miR475b in *Populus suaveolens* that responded to low-temperature stress, including the auxin response element (AuxRR-core), gibberellin response element (GARE), salicylic acid response element (TCA-element), ethylene response element (ERE), and jasmonic acid response element (CGTCA-motif, TGACG-motif) [48,49]. In total, 101 classes of *cis*-acting elements were identified in poplar, including abscisic acid response elements (ABREs), heat stress response elements (HSEs), anaerobic-induced elements (AREs), MYB binding sites, low-temperature-induced response elements (LTRs), chloroplast differentiation elements (HD-Zip 1), leaf shape development elements (HD-Zip 2), and endosperm expression elements (GCN4 and Skn-1 motifs) [33].

From these studies, we can see that different stress-responsive miRNAs in different plants have some *cis*-regulatory elements in common and also share some features. The TC-rich element is present in the promoters of the disease-responsive miRNAs of cassava and *Arabidopsis*. W-box is present in the promoters of multiple stress response miRNAs, including P deficiency in soybean, anthracnose disease in cassava, SO_2_ stress in *Arabidopsis*, and salt stress in rice. The TCA-element is present in the promoter of the miRNA response to cold in rubber trees and the miRNA response to *Bacillus velezensis* FZB42 in *Arabidopsis*. In the same situation as that for the identification of TSSs and promoters of miRNAs, taking advantage of the biotech software, a large number of *cis*-regulatory elements can be obtained through computational methods. In future studies for certain miRNAs, experimental validation, such as the deletion mutation method, can be applied to determine the core elements of miRNA promoters.

## 3. Mechanisms of miRNA Transcriptional Regulation

miRNA transcription is regulated by general and specific transcription factors. Transcription factors can bind to *cis*-acting elements on the promoters of miRNAs to activate the transcription of these miRNAs, which is essential for the spatiotemporal-specific expressions of miRNAs or their adaptation to environmental changes. In addition to transcription factors, several other proteins have been found to regulate miRNA transcription by promoting or repressing miRNA transcription directly or indirectly through binding to RNA polymerase II or the miRNA promoter. At present, multiple transcription factors and other proteins have been identified in several species involved in the regulation of multiple biological processes in plant growth and development (Table 2).

### 3.1. The Involvement of Transcriptional Regulation of miRNA in Plant Growth Processes

In *Arabidopsis*, a B3 transcription factor, FUS3, binds to the promoters of *MIR156A* and *MIR156C* and positively regulates the expression levels of pri-miR156a and pri-miR156c [50]. ABI3, an anabolic acid-insensitive protein in the B3 transcription factor family, promotes *MIR156* expression in early seed development, but represses it in late seed development, which is involved in the regulation of the transition from embryo to seedling [51]. The photomorphogenic transcription factor HY5 negatively regulates the expression of *MIR775a* in the aerial organs of *Arabidopsis* and positively regulates its expression in the roots, participating in the process of cell wall remodeling [52]. In rice, the OsIDD2 protein, containing four zinc finger motifs, binds to the *OsmiR396* promoter to promote the transcription of *miR396* and reduce the expression level of its target gene, GRF [63]. Plants overexpressing *OsIDD2* gene show a dwarf phenotype with a higher expression of *OsmiR396* and a lower expression of *GRF1* [63].

### 3.2. The Involvement of Transcriptional Regulation of miRNA in Plant Leaf Development

The *REVOLUTA (REV)*, *PHABULOSA (PHB)*, and *PHAVOLUTA (PHV)* genes are three *HD-ZIP III* family genes regulating leaf adaxial–abaxial patterning. They are targeted and repressed by abaxially expressed miR165/166 to regulate leaf polarity [66]. However, REV, PHB, and PHV proteins can interact with the HD-ZIP II transcription factors HOMEOBOX ARABIDOPSIS THALIANA 3 (HAT3) and ARABIDOPSIS THALIANA HOMEOBOX 4 (ATHB4) proteins, and the interacting protein complex can bind to conserved *cis*-elements on the *MIR165/166* promoter to repress *MIR165/166* transcription adaxially, which, in turn, represses the expressions of *HD-ZIP III* genes to maintain leaf polarity [53].

### 3.3. The Involvement of Transcriptional Regulation of miRNA in Plant Flower Development

As a target gene of miR172, the class A gene *APETALA2 (AP2)* is downregulated in inner floral whorl organs such as stamens and carpels [54], while in the outer floral whorl organs of *Arabidopsis*, it has been confirmed that AP2 can recruit LUG, a co-repressor protein of SEU, to the *MIR172* promoter through ChIP, BiFC, yeast two-hybrid, and yeast three-hybrid crosses experiments. Moreover, the miR172 expression is significantly upregulated in *lug*, *seu*, and *ap2* mutants, showing that AP2 can interact with the LEUNIG (LEU) and SEUSS (SEU) proteins to repress miR172 transcription [54]. *SPLs* have been found to be target genes of miR156. In mulberry, six SPL transcription factors recognized the promoter of *MIR172* and activated miR172 expression, revealing that *SPL* genes regulating the transcription of miR172 are involved in the flowering development in perennial woody plants [62]. From a study on citrus flower development, miR167a was found to be specifically expressed in the stamen filaments and anthers of pummelo [65]. The DREB transcription factor can bind to and interact with the *MIR167a* promoter to repress its expression by yeast-one hybrid and dual luciferase assays, revealing the regulatory mechanism of *MIR167* and its upstream element in citrus stamen development [65]. In addition to specific miRNA genes, some transcription factors or proteins can generally bind the promoters of multiple miRNAs. Yeast two-hybrid, pull-down fusion protein sedimentation, and immunoblotting experiments have confirmed that the NOT2 (Negative on TATA less2) protein can bind RNA polymerase II to stimulate miRNA transcription and elongation to regulate miRNA biosynthesis [67]. The expressions of multiple miRNA precursors and mature miRNAs (miR158a, miR159a, miR164b, miR167a, and miR168a) were decreased in *not2* mutants of *Arabidopsis*, leading to severe male organogenesis defects, similar to miRNA mutants [67]. Similar to NOT2, the SANT structural domain protein, the PWR protein, can regulate *MIR172* transcription and floral organ development by promoting the accumulation of RNA polymerase in the promoter regions of *MIR172a* and *MIR172b* [55].

### 3.4. The Involvement of Transcriptional Regulation of miRNA in the Synthesis of Secondary Metabolites

miR828 plays an important role in the biosynthesis of the anthocyanins in the peel of apple fruit. The expression of miR828 in this peel is maintained at a comparatively low level during the apple fruit coloration stage and increases rapidly during the late trans-color stage [64]. An overexpression of miR828 in apple and *Arabidopsis* decreases anthocyanin synthesis. Yeast one-hybrid and dual luciferase assays have shown that MdMYB1 binds the miR828 promoter and positively regulates miR828 expression, revealing the involvement of MYB-regulated MIR828 transcription in the biosynthesis mechanism of plant fruit anthocyanins [64].

### 3.5. The Involvement of Transcriptional Regulation of miRNA in Plant Disease Resistance

The *TPR1 (transcriptional corepressor1)* gene is a transcriptional repressor of an *NBS-LRR* gene encoding the disease-resistance protein SNC1 (Suppressor of npr1-1). An overexpression of the *TPR1* gene causes reductions in the levels of several pri-miRNAs and miRNAs (miR164, miR173, miR319, miR390, and miR159) [68]. As a negative regulator of SNC1, the F-box protein CPR1 can mediate the degradation of the SNC1 protein. The *cpr1aba1* mutant results in a large accumulation of the SNC1 protein in the nucleus, causing transcriptional reductions in several miRNAs (pri-miR159a, pri-miR159b, pri-miR164b, pri-miR166a, and pri-miR167a). Since miRNAs (miR173 and miR390, etc.) can target and trigger some disease-resistance genes to produce phasiRNAs, the *cpr1aba1* mutant causes a reduction in the abundance of phasiRNAs produced on four disease-resistance genes, resulting in an upregulation of the expressions of 70 resistance genes. Therefore, miRNA genes are transcriptionally regulated by the *SNC1* gene to participate in plant resistance to pathogens [68].

In addition to the disease resistance originating from the endogenous miRNA targeting and regulation on resistant genes, artificial miRNAs (amiRNAs) and miRNA-induced gene silencing (MIGS) have recently become miRNA-based strategies for obtaining pest and disease resistance [2,69]. Artificial microRNAs (amiRNA) are generally designed from an endogenous miRNA precursor (pre-miRNA), which is used as a structural support in which the miRNA:miRNA* is replaced with a specific miRNA complementary to the desired target sequence [70]. The MIGS approach exploits a special 22-nuclotide miRNA of *Arabidopsis thaliana*, miR173, which can trigger the production of *trans*-acting small RNAs [71]. Different from the miRNA transcription on their own promoter, pre-amiRNAs and the MIGS vector are generally constructed using a plasmid containing an effective constitutive-like *35S* promoter to mediate the targeted viral RNA cleavage to confer resistance to various diseases, such as the Cassava brown streak virus (CBSV), Ugandan cassava brown streak virus (UCBSV), cotton leaf hopper (*Amrasca biguttula*), cotton whitefly (*Bemisia tabaci*), and so on [2,69].

### 3.6. The Involvement of the Transcriptional Regulation of miRNA in Plant Abiotic Stress

Environmental stresses (such as saline, nutrient deficiency, and heavy metal) greatly constrain normal plant growth and development [72,73,74,75,76]. miRNAs are involved in various abiotic stresses, including salinity, drought, heat, cold, nutrient deficiency, oxidative stress, UV radiation, heavy metal toxicity, and so on [77]. In recent years, the regulation mechanism of miRNAs in the abiotic response has been discovered in some plants. In *Arabidopsis* and tomato, the HSF transcription factor is involved in heat stress tolerance through binding to the *MIR169* promoter to positively regulate the transcription of *MIR169* and negatively regulate the expression of its target gene *NF-YA9/10* [61]. The SERRATE protein, a conserved RNA processing factor in eukaryotes, encodes a C2H2 zinc finger protein. The SERRATE protein can regulate the drought tolerance in apple by positively regulating the transcription of *MIR399* and negatively regulating the transcriptions of *MIR166*, *MIR172*, and *MIR319* [51]. In rice, a Calmodulin-binding Transcription Activator (OsCAMTA4) binds to the promoters of *MIR156* and *MIR167h* to activate the expressions of two miRNAs, participating in the plant response to abiotic stress [62]. In addition to being part of the RNA-silencing complex to cleave mRNA in the cytoplasm, AGO1 also plays roles in miRNA biogenesis at the transcriptional level in the nucleus. From an immunoprecipitation analysis, it was found that AGO1 can bind to the chromatin of miR161 and miR173. In the *ago1* mutant, the expression levels of miR161 and miR173 markedly decreased under sanity stress [56].

Many miRNAs are responsive to environmental signals. The miR156 regulating network is involved in plant adaptation to shade. In shade conditions, the bHLH class protein PHOTOCHROME-INTERACTING FACTORS (PIFs) can bind five *MIR156* promoters, repress their expressions, and concomitantly enhance the expressions of *SPL* family genes to mediate the plant’s shade response syndrome (SAS) [57]. The leucine zipper (bZIP)-like transcription factor HY5 (ELONGATED HYPOCOTYL5) can bind to two G/C box elements on the promoter *MIR163* and positively regulate its expression in response to light signals, in order to promote primary root elongation in seedlings without affecting lateral root growth [58].

The MYB transcription factor in *Arabidopsis thaliana* can activate miR399 expression and reduce the expression of its target gene *UBC (ubiquitin-conjugating enzyme)* by binding to the MYB binding site on the *MIR399* promoter in response to phosphate starvation [59]. The restricted expression of UBC relieves the inhibition of the phosphorus transporters by UBC to promote phosphorus uptake and transport [59].

### 3.7. The Involvement of Transcriptional Regulation of miRNA in Phytohormone Signaling Pathways

Plant hormones play important roles in plant growth and development [78,79,80]. Several members of the soybean *MIR160* and *MIR167* families contain multiple auxin response factor (ARF)-binding elements and gibberellin response factor (GARF)-binding elements in their promoter regions, suggesting that ARF and GARF transcription factors may bind to the promoters of *MIR160* and *MIR167* to regulate their expressions. Since ARF transcription factor family members such as *ARF17*, *ARF18*, *ARF6*, and *ARF8* are target genes of both miR160 and miR167, a potential feedback regulatory mechanism between miR160 and miR167 and the ARF- and GRF-binding elements was revealed [37]. EIN2 and EIN3 are important transcription factors in the ethylene signaling pathway. An overexpression of *EIN2* in *Arabidopsis* elevates the expression level of miR397b/miR857, as well as reduces the expressions of the target genes *LAC4* and *LAC17*, resulting in a significant reduction in the lignin accumulation in vascular bundles [60]. Yeast one-hybrid experiments have confirmed that EIN2 and EIN3 can bind to ethylene response factors (ERFs) on the *MIR397b*/*MIR857* promoter to promote transcription, revealing the molecular mechanisms of the miRNAs involved in the regulation of plant lignin synthesis in response to ethylene signaling [60].

### 3.8. miRNA Transcriptional Regulation Mediated by General Transcription Factors

**Figure 2:**
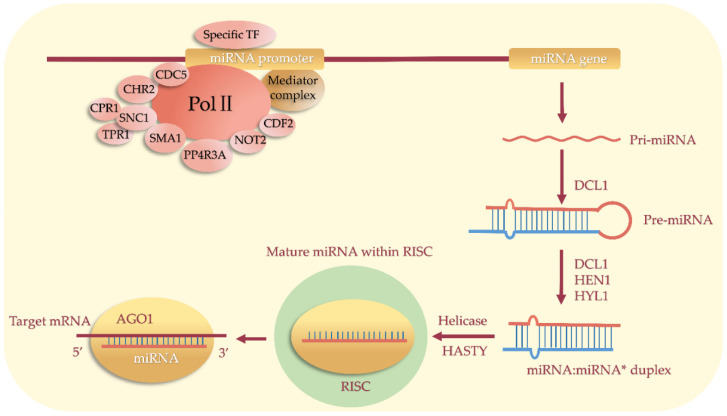
The transcriptional regulation of miRNA by the general transcription factors. *DCL1 (DICER-LIKE 1)*, *HYL1 (methyltransferase HUA ENHANCER 1)*, *HEN1 (HUA ENHANCER 1)*, *AGO1 (ARGONAUTE1)*, *RISC (RNA-induced silencing complex)*, *TPR1 (transcriptional corepressor1)*, *SNC1 (suppressor of npr1-1*, *constitutive 1)*, *CPR1 (constitutive expresser of PR genes)*, *CDF (Dof transcription factor)*, *SMA1 (Prp28 homolog SMALL 1)*, *CHR (chromatin remodeling factor)*, *CDC5 (CELL DIVISION CYCLE 5)*, *PP4R3A (phosphatase protein PP4)*, *NOT2 (NEGATIVE ON TATA LESS 2)*, *and TF (transcription factor)*.

The transcription of miRNA genes requires the participation of a mediator complex, which can not only recruit RNA polymerase II during transcription, but also interact with specific transcription factors (Figure 2). This mediator complex can bind directly to the miRNA promoters in the presence of some activating proteins to initiate miRNA genes’ transcription (Figure 2). In the mediate complex *med20a* mutant, the expressions of six detected miRNA precursors (pri-miR159, pri-miR163, pri-miR165a, pri-miR166a, pri-miR167a, and pri-miR171a) were downregulated by 20%–70% compared to the control and caused abnormal phenotypes such as smaller plants, shorter petioles, the downward bending of leaves, late flowering, and reduced fertility [81,82]. CDC5 (cell division cycle 5), a kind of conserved DNA-binding protein widely found in eukaryotes, is involved in various processes of plant development through binding to the Pol II promoters of multiple miRNAs and then regulating miRNA transcription [83]. In *Arabidopsis cdc5* mutants, several miRNAs (miR166/165, miR167, miR159/319, miR390, miR171, miR172, miR173, miR156, and miR163) were significantly downregulated, resulting in various developmental defects, including plant size, leaf shape, delayed flowering, and sterility [83]. CDF is a kind of DNA-binding with one finger family protein [84]. *Arabidopsis* CDF2 can bind to miRNA promoters to promote or repress miRNA transcription [85]. In the *cdf2* mutant, two miRNA precursors (pri-miR172a and pri-miR394a) were significantly upregulated, and 17 other miRNA precursors were significantly downregulated [85]. PP4R3A, a regulatory subunit of the phosphatase protein PP4, can bind to Pol II and recruit the polymerase to the promoter regions of miRNAs, thereby promoting miRNA transcription and enhancing the expressions of multiple miRNAs [86]. In addition, SMA1, a homolog of the Prp28 spliceosomal protein, is also required for miRNA transcription [87]. A mutation of the *SMA1* gene resulted in a significant downregulation of 11 miRNA precursors and mature miRNAs and a significant decrease in the Pol II bound to the promoter regions of five miRNAs [87]. In addition, an ATPase chromatin remodeling factor CHR2 and transporter HST can also bind to Pol II to promote the accumulation of polymerase at the miRNA promoter regions [58,87,88].

Currently, upstream regulatory elements have been focused on Arabidopsis and few have been identified in other plants. From the present studies, for the same conserved miRNAs, the upstream transcription factors of miRNAs are probably totally different in different developmental stages, such as miR156 in seed development and in leaves (Table 2). And the upstream transcription factors of a same miRNA are probably different in different plant species, such as miR399 in *Arabidopsis* and in apple, demonstrating that miRNAs are tissue-specifically and species-specifically regulated by transcription factors through combining with different cis-elements. Although the mechanisms of miRNA transcriptional regulation are complex, many effective identification methods, such as yeast hybridization, EMSA, ChIP, BiFC, and transgenic, etc., have been shown in previous research and provide a reference for applications in the other miRNAs of different plants.

## 4. Summary and Prospects

Several studies have been conducted on the transcriptional regulation of miRNA, including multiple miRNAs in model plants and some woody plants, which are involved in multiple biological processes such as plant growth and development, stress response, and signal transduction, etc. However, compared to the number of identified miRNAs, there is still a lack of analysis of the upstream regulators and transcriptional regulation mechanism of miRNA genes. Until now, the transcription factors for only a small number of conserved miRNAs have been demonstrated. How some other conserved and species-specific miRNAs are regulated at the transcriptional level is still unknown. With more and deeper research on the miRNA function in different plants, future research on miRNA promoters and transcriptional regulatory mechanisms will be more extensive. Researchers can identify miRNA promoters and functional elements using 5’ RACE, the GUS staining activity assay, and other techniques, and identify more miRNA upstream regulatory factors using yeast hybridization, EMSA, ChIP, BiFC, and transgenic, etc., to further enrich the study of plant miRNA regulatory mechanisms from upstream to downstream and provide new valuable functional elements and genetic resources for a plant’s genetically engineered traits improvement.

## Figures and Tables

**Table 1:** Genomic identification methods of microRNA promoters.

Species	Counts of miRNAs Loci	Counts of Identified miRNAs Promoters	Identification Methods	Reference
Arabidopsis	52	63	5’ RACE	[30]
Arabidopsis	95	98	Common query voting (CoVote)	[26]
Rice	114	104	CoVote	[26]
Rice	158	249	TSSP	[32]
Soybean	22	64	TSSP	[35]
Soybean	12	191	TSSP	[34]
*Populus*	139	229	TSSP	[33]
Soybean	440	699	Degradome libraries and TSSP	[37]
Soybean	298	132	Genome-wide profiles of nine histone markers	[36]
Arabidopsis	40	34	Query-Ranked Frequent Rule (QRFR)	[39]
Cassava	23	21	PromPredict and TSSP	[38]

**Table 2:** Summary of upstream transcription factors of miRNAs.

Organism	miRNA	Upstream Transcription Factors of miRNAs	Positive or Negative Regulation of miRNA	Functions of the Modules	Reference
Arabidopsis	miR156	FUS3	Positive	Seed development	[50]
	miR156	ABI3	Positive and negative	Positive regulation in early seed development but negative regulation in late seed development	[51]
	miR775	HY5	Positive and negative	Cell wall remodeling, positive regulation in root growth but negative regulation in aerial organs development	[52]
	miR165/166	HD-ZIP II and III family genes	Negative	Leaf development	[53]
	miR172	AP2, LUG, SEU	Negative	Flower development	[54]
	miR172	PWR	Positive	Flower development	[55]
	miR161 and miR173	AGO1	Positive	Salinity response	[56]
	miR156	PIFs	Negative	Shade response	[57]
	miR163	HY5	Positive	Light response	[58]
	miR399	MYB	Positive	Phosphate starvation response	[59]
	miR160 and miR167	ARF and GARF	Positive	Auxin response	[37]
	miR397b/miR857	EIN2 and EIN3	Positive	Lignin synthesis in response to ethylene signaling	[60]
Arabidopsis and tomato	miR169	HSF	Positive	Heat stress	[61]
Rice	miR156 and miR167h	OsCAMTA4	Positive	Drought response	[62]
Rice	miR396	IDD2	Positive	Stem elongation	[63]
Apple	miR399	SERRATE	Positive	Drought response	[63]
Apple	miR166, miR172 and miR319	SERRATE	Negative	Drought response	[51]
Apple	miR828	MdMYB1	Positive	Anthocyanin synthesis	[64]
Pummelo	miR167a	DREB	Negative	Flower development	[65]
Mulberry	miR172	SPLs	Positive	Flower development	[62]
